# The Altered Reconfiguration Pattern of Brain Modular Architecture Regulates Cognitive Function in Cerebral Small Vessel Disease

**DOI:** 10.3389/fneur.2019.00324

**Published:** 2019-04-05

**Authors:** Renyuan Liu, Haifeng Chen, Ruomeng Qin, Yucheng Gu, Xin Chen, Junhui Zou, YongCheng Jiang, Weikai Li, Feng Bai, Bing Zhang, Xiaoying Wang, Yun Xu

**Affiliations:** ^1^Department of Neurology, Drum Tower Hospital, Medical School and The State Key Laboratory of Pharmaceutical Biotechnology, Institute of Brain Science, Nanjing University, Nanjing, China; ^2^Jiangsu Province Stroke Center for Diagnosis and Therapy, Nanjing, China; ^3^Nanjing Neuropsychiatry Clinic Medical Center, Nanjing, China; ^4^College of Computer Science and Technology, Nanjing University of Aeronautics and Astronautics, Nanjing, China; ^5^Department of Radiology, Drum Tower Hospital, Medical School of Nanjing University, Nanjing, China; ^6^Departments of Neurology, Harvard Medical School, Massachusetts General Hospital, Charlestown, MA, United States

**Keywords:** small vessel disease, cognitive impairment, network reconfiguration, compensation, visuospatial processing

## Abstract

**Background:** Cerebral small vessel disease (SVD) is a common cause of cognitive dysfunction. However, little is known whether the altered reconfiguration pattern of brain modular architecture regulates cognitive dysfunction in SVD.

**Methods:** We recruited 25 cases of SVD without cognitive impairment (SVD-NCI) and 24 cases of SVD with mild cognitive impairment (SVD-MCI). According to the Framingham Stroke Risk Profile, healthy controls (HC) were divided into 17 subjects (HC-low risk) and 19 subjects (HC-high risk). All individuals underwent resting-state functional magnetic resonance imaging and cognitive assessments. Graph-theoretical analysis was used to explore alterations in the modular organization of functional brain networks. Multiple regression and mediation analyses were performed to investigate the relationship between MRI markers, network metrics and cognitive performance.

**Results:** We identified four modules corresponding to the default mode network (DMN), executive control network (ECN), sensorimotor network and visual network. With increasing vascular risk factors, the inter- and intranetwork compensation of the ECN and a relatively reserved DMN itself were observed in individuals at high risk for SVD. With declining cognitive ability, SVD-MCI showed a disrupted ECN intranetwork and increased DMN connection. Furthermore, the intermodule connectivity of the right inferior frontal gyrus of the ECN mediated the relationship between periventricular white matter hyperintensities and visuospatial processing in SVD-MCI.

**Conclusions:** The reconfiguration pattern of the modular architecture within/between the DMN and ECN advances our understanding of the neural underpinning in response to vascular risk and SVD burden. These observations may provide novel insight into the underlying neural mechanism of SVD-related cognitive impairment and may serve as a potential non-invasive biomarker to predict and monitor disease progression.

## Introduction

Cerebral small vessel disease (SVD) is a significant contributor to cognitive dysfunction (1). It is characterized by white matter hyperintensities (WMH), lacunar infarcts (LI), microbleeds and the Virchow-Robin space in MRI (1). Although the mechanism is still incompletely understood, SVD is generally considered to be the result causes of aging and vascular risk factors including hypertension, diabetes, and smoking (2). More vascular risk factors create an easier path to SVD (3). The Framingham Stroke Risk Profile (FSRP) is a composite risk index of vascular risk factors and has been used to identify the population at high risk for SVD (4, 5).

Early identification of individuals at risk for cognitive decline is important to the development of effective therapies for cognitive decline or dementia in SVD. Previous publications have indicated that the progression or location of WMH could induce cognitive decline (6, 7). Currently, a promising brain functional imaging technique, that is, resting-state fMRI has been widely used in the human brain functional network researches, which can show the metabolism in the different areas, spontaneous activity in different mode regions, and intra- or inter-regional connectivity among different brain networks (8).

Functional connectivities are not homogeneously distributed across the whole network, but gather into subnetworks (i.e., modules) that are densely connected internally but only weakly coupled externally (8). Overall, modular organization may be conductive to the greater robustness and adaptability of the brain network responding to internal and external changes (8). Previous studies have observed modular reconfiguration of brain networks. In patients with subcortical vascular mild cognitive impairment, the executive control network (ECN) module was notably rearranged; i.e., the posterior parietal regions were separate from ECN as a new module (9). A gene-connectome study demonstrated that APOE ε4 in patients with Alzheimer's disease led to the reconfiguration of the posterior default mode network (pDMN) and ECN correlated with cognitive performance (10). Furthermore, a task-state MRI study revealed that normal individuals showed dynamic integration between specialized brain modules at different cognitive loads (11). Therefore, modularity analysis could provide further insights into the SVD-related cognitive impairment.

In this study, we applied graph-theoretical modularity analysis to resting-state functional MRI data and characterized the brain modular network organization in subjects with SVD or those at risk. Furthermore, we explored the relationship among SVD burden, modular measures and cognitive performance. We hypothesize that reconfiguration of modular architecture emerges during the progression of SVD and mediates the relationship between SVD burden and cognitive function.

## Materials and Methods

### Participants

This is hospital-based Cross-study (Clinical Trial: ChiCTR-OOC-17010562), which consists of 85 Han Chinese participants (49 SVD subjects and 36 matched healthy controls [HC]) aged between 50 and 80 years. SVD divided into SVD-non cognitive impairment (SVD-NCI, *n* = 25) and SVD-mild cognitive impairment (SVD-MCI *n* = 24) based on neuropsychological assessment. HC was split up into HC-low risk (risk <15%, *n* = 17) and HC-high risk (risk >15%, *n* = 19), following Stroke Risk Prediction Model (12). SVD criteria was defined by the presence on neuroimaging: WMH (Fazekas scale 2 or higher) with or without lacunar infarct (13, 14). Exclusion criteria included intracranial hemorrhage; non-SVD-related WMH mimics (e.g., multiple sclerosis); cardioembolic source (e.g., atrial fibrillation); intra/extracranial large artery stenosis >50%; dementia [Mini-Mental State Examination (MMSE) ≤23] and other neurological or psychiatric disorders (15). This research was approved by the Ethics Committee of Nanjing Drum Tower Hospital, and signed informed consent was obtained from all participants.

### Stroke Risk Prediction Model

FSRP is a clinical and composite risk score of vascular risk factors that predicts 10-year probability of stroke for individuals who are free of stroke at baseline (12). This model is based on the following risk factors: age, systolic blood pressure, use of hypertensive medication, diabetes mellitus, cigarette smoking, atrial fibrillation, cardiovascular heart disease, and left ventricular hypertrophy. A higher FSRP indicates a higher risk of developing a stroke event (12). The score ranges from 1 to 27 points for women and 1–30 points for men. In this study, participants with atrial fibrillation were excluded due to cardioembolic source. So, we excluded points assigned for atrial fibrillation. The sex-specific score is then converted to 10-year probability of strokes ranging from 1 to 84% for women and 3–88% for men (12).

### Neuropsychological Assessment

All participants underwent a standardized neuropsychological evaluation protocol, which included the general cognitive examination and multiple cognitive domain assessments performed by an experienced neuropsychologist. General cognitive function was evaluated by MMSE and Beijing version of the Montreal Cognitive Assessment (MoCA-BJ). In this study, we used MoCA-BJ to detect SVD-MCI. Since education is the strongest non-cognitive factor influencing the assessment of MoCA-BJ, the optimal cutoff points are determined according to education level (or years of education). For subjects with no formal education, the MoCA-BJ cutoff was 13/14; for subjects with 1–6 years of education, the MoCA-BJ cutoff was 19/20; and for subjects with 7 or more years of education, it was 24/25. The raw examination scores were transformed to Z-scores so as to calculate each cognitive domain performance. Episodic memory is a compound score that includes the mean of the Z-scores of Auditory Verbal Learning Test-delayed recall (AVLT-DR) and Wechsler Memory Scale Visual Reproduction-delayed recall (WMS-VR-DR). Visuospatial function (VPF) was calculated as the mean of the Z-scores of Clock Drawing Test (CDT) and Visual Reproduction-copy (VR-C). Information processing speed (IPS) is a compound score of the average Z-scores of Trail Making Test-A (TMT-A), Stroop Color and Word Tests A and B (Stroop A and B). Language consisted of Category Verbal Fluency (CVF) and Boston Naming Test (BNT). Executive Function was calculated as the average Z-scores of Digit Span Test-backward (DST-backward), TMT-B, and Stroop C.

### MRI Scanning

All of the subjects were scanned by a Philips 3.0-T scanner (Philips Medical Systems, The Netherlands) with a homogeneous birdcage head coil in order to reduce head movements. Prior to the scan, all subjects were instructed to keep their eyes closed but not fall asleep, think of nothing, and move as little as possible during data acquisition. Finally, a simple questionnaire indicated that all of the subjects had not fallen asleep during the scan. The high-resolution T1-weighted sagittal images covering the whole brain acquired by turbo fast echo acquisition as follows: repetition time (TR) = 9.8 ms, echo time (TE) = 4.6 ms, flip angle (FA) = 8°, acquisition matrix = 256 × 256, number of slices = 192, thickness = 1.0 mm, FOV = 250 × 250 mm^2^. The 3D fluid-attenuated inversion recovery (FLAIR) images were acquired by the sequence: TR = 4,500 ms, TE = 333 ms, time interval (TI) = 1,600 ms, acquisition matrix = 270 × 260, voxel size = 0.95 × 0.95 × 0.95 mm^3^, number of slices = 200. The resting-state functional scans covering 230 volumes were obtained with a gradient-recalled echoplanar imaging sequence: TR = 2,000 ms, TE = 30 ms, FA = 90°, acquisition matrix = 64 × 64, number of slices = 35, thickness = 4.0 mm, FOV = 240 × 240 mm^2^. WMH automated segmentation and volume quantification was processed in the Wisconsin White Matter Hyperintensities Segmentation Toolbox version 1.3 (W2MHS v1.3, https://sourceforge.net/projects/w2mhs) based on FLAIR and T1 images. The total WMH included periventricular-WMH (PWMH) and deep-WMH (DWMH). Intracranial volume was calculated as a sum of gray matter (GM), white matter and cerebrospinal fluid volume using automated segmentation on T1 images in Statistical Parametric Mapping (SPM8, http://www.fil.ion.ucl.ac.uk /spm). WMH volume was normalized to the intracranial volume (16). Lacunes of presumed vascular origin were defined as hypointense areas (>3 mm and ≤15 mm in diameter) on FLAIR and T1 images, distinguished from enlarged perivascular spaces and infraputaminal pseudolacunes (1). Lacunes were counted by two trained raters blinded to the participants' clinical information.

### Image Preprocessing

The resting-state fMRI data was preprocessed by the Graph Theoretical Network Analysis Toolbox version 2.0 (GRETNA v2.0, http://www.nitrc.org/projects/gretna/) based on SPM8. After removing the first 10 volumes, the remaining functional images were corrected for intravolume time offsets and intervolume geometrical displacements. No subjects performed a displacement >2 mm or an angular rotation >2° in any direction. Next, the obtained images were spatially normalized to the Montreal Neurological Institute (MNI) space and resampled to 3 × 3 × 3 mm voxels. The resulting images were further band-pass filtered within the frequency range of 0.01–0.08 Hz to reduce the low-frequency drift and high frequency physiological noise (17). Linear trends were also removed. Finally, several nuisance signals were regressed out, including the Friston 24-motion parameter model (six head motion parameters, six head motion parameters one time point before, and the 12 corresponding squared items), global mean, white matter and cerebrospinal fluid signals (18).

### Network Construction

In this study, functional brain networks were constructed at the large-scale level with nodes for brain regions and edges for interregional functional connectivity (FC). To define the network nodes, we divided the brain into 1024 contiguous and uniform regions of interest (ROIs) based on a high resolution, randomly partitioning brain atlas (19). To define network edge, we calculated Pearson correlation coefficients for each pair of 1024 ROIs between the regional mean time series. To improve the normality, these correlation coefficients were translated to z values by Fisher's r-to-z transform. We restricted our analysis to positive correlations because of the ambiguous interpretation of negative correlations (20). As described in the previous study, brain networks were not fully connected at lower sparsity threshold and were less likely to remain small-world architecture at higher sparsity threshold (11). In this study, the matrix was thresholded at a set of sparsity (ranging from 0.10 to 0.30, with steps of 0.01) to obtain a binary undirected network (21).

### Modularity

A module is referred to as a collection of nodes that are densely connected with each other but less connected with other nodes. And the modularity *Q* of a network quantifies the efficacy of segmenting a network into modules, which was defined as follows:

(1) $$\begin{array}{l} Q=\overset{Nm}{\underset{i=1}{\sum }}\left[li/L-{\left(di/2L\right)}^{2}\right] \end{array}$$

where *N*_*m*_ is the number of modules, *L* is the total number of edges in the network, *l*_*i*_ is the number of within-module edges in the module *i* and *d*_*i*_ is the sum of the linked edges at each node in the module *i*. In this study, we used a spectral optimization algorithm to detect the modular community structure, which was proposed by Newman (22). In practice, the network modularity *Q* with a powerful modular structure typically ranges from 0.3 to 0.7 (23). Given that the sparsity threshold could have an effect on modular partitioning, we performed the modularity analysis on group-level brain networks, applying a threshold of 20% sparsity at each group (9). According to prior investigations associated with cognition (11, 24), DMN and ECN modules, which were identified from the module partitioning at each group by visual inspection, were of particular interest in our study. Notably, to ensure comparability, we apply the module partitioning of HC-low risk group as the unified standard in the following analyses at module and nodal levels.

At the module level, we measured intramodule connectivity density (*D*_*s*_) and intermodule connectivity density (*D*_*s,t*_) as follows:

(2) $$\begin{array}{l} Ds=\frac{2\underset{i,j\in s}{\sum }\varepsilon i,j}{Ns\left(Ns-1\right)} \end{array}$$

where *N*_*s*_ is the number of nodes within module *s*, and ε_*i,j*_ are the existing edges within module *s*.

(3) $$\begin{array}{l} Ds,t=\frac{\underset{i\in s,j\in t}{\sum }\varepsilon i,j}{Ns*Nt} \end{array}$$

where *N*_*s*_ is the number of nodes within module *s* and *N*_*t*_ is the number of nodes within module *t*, and ε_*i,j*_ are the existing edges between module *s* and module *t*.

At the nodal level, within-module degree (WD) and participation coefficient (PC) were calculated as follows (25):

(4) $$\begin{array}{l} WDi=\frac{ei-ēs}{\sigma s} \end{array}$$

where *e*_*i*_ is the nodal degree of a node *i* within module *s* and ē*s* is the average nodal degree of all nodes in module *s*, and σ_*s*_ is the standard deviation of the within module nodal degree of all nodes in module *s*.

(5) $$\begin{array}{l} PCi=1-{\overset{Nm}{\underset{s=1}{\sum }}\left(\frac{ki,s}{ki}\right)}^{2} \end{array}$$

Where *N*_*m*_ is the number of modules and *k*_*i,s*_ is the number of connections between the node *i* and module *s*. *k*_*i*_ is the total number of connections of node *i* to all other nodes in the *N*_*m*_ modules.

### Statistical Analysis

Differences between groups in demographic, neuroimaging characteristics and cognitive assessment were analyzed using a Chi-squared (χ^2^) test or one-way analysis of variance (ANOVA) in SPSS version 22 (IBM Corp., Armonk, NY). The significance level was set at *P* < *0.05*.

For module level metrics, we used ANOVA to investigate whether there were significant group differences in modularity, intramodule connectivity density and intermodule connectivity density. The significant level was set at *P* < *0.05*. For nodal-wise measures (i.e., WD and PC), we applied GRETNA to investigate the significantly different brain regions between groups, and false discovery rate (FDR) was performed at an α level of 0.01 to correct for multiple comparisons. Then, a *post hoc* test was used to determine the change pattern of nodal-wise metrics in differential regions. In all analyses, age, gender, education level, GM volume, and number of lacunes or WMH volume were controlled for as confounding covariates.

To investigate the relationship among MRI markers, network metrics and cognitive performance, a multiple regression analysis and mediation analysis were performed by using SPSS while controlling for relevant covariates (age, sex, education level, GM, and number of lacunes or WMH volume).

## Results

### Demographic and Clinical Characteristics

Demographic and clinical data for the HC subgroups (HC-low risk and HC-high risk) and SVD subgroups (SVD-NCI and SVD-MCI) are summarized in Table 1. There were no significant differences in gender and years of education between four groups. However, HC-low risk group showed significantly lower age compared with other groups. In subsequent analyses, we controlled for the age as a confounding covariate. WMH and PWMH volume significantly differed among groups (*P* < 0.001). Both of SVD subgroups had a higher WMH and PWMH volume compared to each HC subgroup. The SVD-MCI group exhibited poorer performances on MoCA-BJ (*P* < 0.001), episodic memory (*P* < 0.001), VPF (*P* = 0.017), IPS (*P* = 0.002), language function (*P* = 0.028) and executive function (*P* < 0.001) than other groups (details of cognitive domain assessment in Table 1).

**Table 1:** Demographic and neuropsychological data.

**Items**	**HC**		**CSVD**		***F/χ2/H***	***p***
	**Low-risk (*n* = 17)**	**High-risk (*n* = 19)**	**NCI (*n* = 25)**	**MCI (*n* = 24)**		
**DEMOGRAPHICS**						
Age (years)	55.47 ± 4.23	68.16 ± 5.32	64.52 ± 10.65	65.92 ± 9.11	8.260	<0.001^b^^*^
Education (years)	11.47 ± 4.09	11.84 ± 3.63	11.16 ± 4.11	12.67 ± 3.38	0.694	0.559^b^
Gender (male/female)	7/10	14/5	12/13	11/13	–	0.179^a^
**NEUROIMAGING CHARACTERISTICS**						
GMV(cm^3^)	540.88 ± 56.93	541.77 ± 46.88	539.75 ± 38.88	538.08 ± 51.44	0.023	0.995^b^
WMV(cm^3^)	505.66 ± 53.69	467.19 ± 52.91	460.83 ± 44.00	463.95 ± 59.47	0.709	0.550^b^
WMH(mm^3^)	470.97 (184.91, 651.34)	719.00 (147.29, 892.00)	2978.46 (762.74, 4019.20)	4826.16 (760.99, 5639.67)	20.220	<0.001^c^^*^
PVWMH	287.38 (111.46, 500.42)	547.20 (107.91, 786.85)	2091.44(468.66, 2913.88)	3633.38 (317.13, 4862.83)	21.266	<0.001^c^^*^
DWMH	97.93 (31.34, 173.13)	41.25 (17.50, 283.61)	298.69 (54.30, 980.61)	431.76 (14.55, 1149.31)	6.056	0.109
Lacunes, number (%)	–	–	7 (28%)	13 (54%)	–	–
**GENERAL COGNITION**						
MMSE	28.71 ± 1.26	28.58 ± 1.39	28.44 ± 1.29	27.75 ± 2.07	1.624	0.19^b^
MoCA-BJ	25.47 ± 0.60	25.73 ± 0.54	26.10 ± 0.45	21.41 ± 0.46	21.789	<0.001^b^^*^
**COMPOSITION Z SCORES OF EACH COGNITIVE DOMAIN**						
Episodic memory	0.60 ± 0.54	−0.10 ± 0.56	0.11 ± 0.54	−0.53 ± 0.91	9.762	<0.001^b^^*^
AVLT-DR	6.53 ± 1.46	5.47 ± 1.47	5.52 ± 1.85	3.75 ± 2.05	9.061	<0.001^b^^*^
VR-DR (WMS)	8.65 ± 3.26	6.37 ± 2.79	7.08 ± 2.68	5.83 ± 3.58	2.964	0.037^b^^*^
Visuospatial processing function	0.18 ± 0.24	0.14 ± 0.21	0.26 ± 0.18	−0.503 ± 0.18	3.584	0.017^b^^*^
CDT	3.96 ± 0.16	3.82 ± 0.14	3.99 ± 0.12	3.35 ± 0.12	5.529	0.002^b^^*^
VR-C	13.71 ± 0.46	13.91 ± 0.41	13.88 ± 0.34	12.98 ± 0.35	1.514	0.217^b^
Information processing speed	0.36 ± 0.75	0.09 ± 0.89	0.18 ± 0.78	−0.51 ± 0.58	5.538	0.002^b^^*^
TMT-A	50.27 ± 5.74	51.15 ± 5.15	46.30 ± 4.27	68.67 ± 4.40	5.014	0.003^b^^*^
Stroop A	16.51 ± 2.15	14.63 ± 1.93	17.66 ± 1.60	24.14 ± 1.65	5.749	<0.001^b^^*^
Stroop B	20.45 ± 2.25	23.25 ± 2.02	19.97 ± 1.67	25.18 ± 1.72	1.816	0.151^b^
Language	0.26 ± 0.20	0.12 ± 0.18	0.12 ± 0.15	−0.4 ± 0.15	3.207	0.028^b^^*^
CVF	17.40 ± 1.01	17.17 ± 0.91	17.43 ± 0.75	15.55 ± 0.77	1.279	0.287^b^
BNT	52.31 ± 1.62	50.77 ± 1.45	50.33 ± 1.20	46.79 ± 1.24	2.999	0.036^b^^*^
Executive function	0.31 ± 0.53	0.36 ± 0.82	−0.12 ± 0.64	−0.38 ± 0.56	6.437	<0.001^b^^*^
DST-backward	5.29 ± 0.38	5.69 ± 0.35	4.80 ± 0.29	4.66 ± 0.29	2.231	0.091^b^
TMT-B	81.39 ± 12.06	79.22 ± 10.83	107.13 ± 8.98	131.21 ± 9.25	6.124	<0.001^b^^*^
Stroop C	29.33 ± 2.67	28.90 ± 2.39	33.47 ± 1.98	36.77 ± 2.04	2.885	0.041^b^^*^

*Values are presented as the mean ± standard error (SE), median (interquartile ranges) or number (percentage)*.

a *the p-value was obtained by χ2 test*.

b *the p-value was obtained by one-way ANOVA and c the p-value was obtained by Kruskal-Wallis one-way ANOVA*.

* *indicates a statistical difference between groups, p < 0.05*.

*HC, health control; CSVD, cerebral small vessel disease; NCI, non-cognitive impairment; MCI, mild cognitive impairment; GMV, gray matter volume; WMV, white matter volume; WMH, white matter hyperintensities. PVWMH, periventricular-white matter hyperintensities; DWMH, deep-white matter hyperintensities; MMSE, mini mental state examination; MoCA-BJ, beijing version of the montreal cognitive assessment; AVLT-DR, auditory verbal learning test-delayed recall; VR-DR, visual reproduction-delay recall; WMS, wechsler memory scale; CDT, clock drawing test; VR-C, visual reproduction-copy; CVF, category verbal fluency; BNT, Boston Naming Test; DST, digit span test; TMT-A and TMT-B, trail making test-A and B; Stroop A, B and C, stroop color and word tests A, B, and C*.

### Brain Module Identification

**Figure 1:**
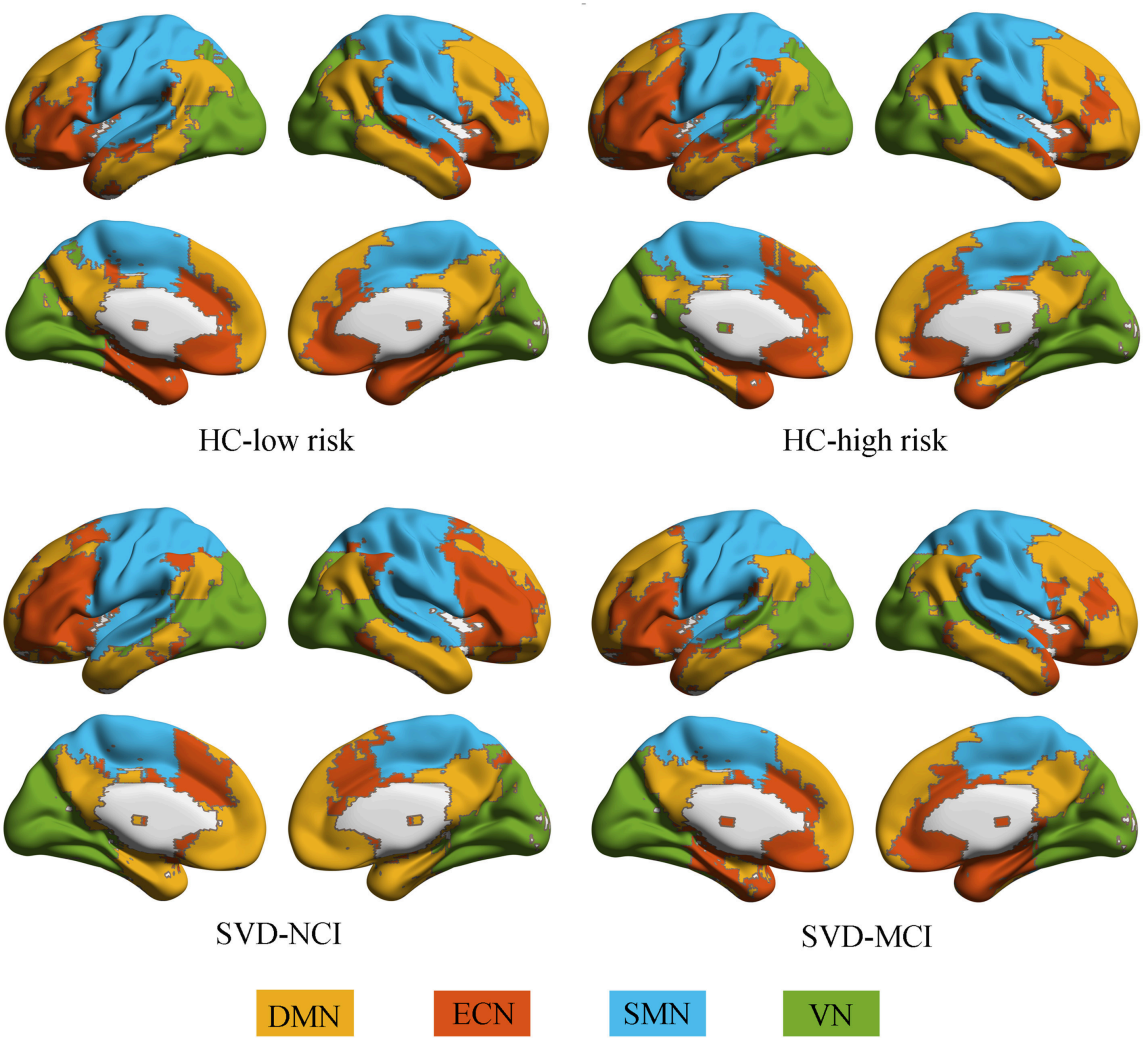
Modular architecture for each group. In each group, four modules were found in the mean functional brain network: the default mode network (yellow), the executive control network (orange), the sensorimotor network (blue), and the visual network (green). HC, healthy control; SVD, small vessel disease; NCI, non-cognitive impairment; MCI, mild cognitive impairment; DMN, default mode network; ECN, executive control network; SMN, sensorimotor network; VN, visual network.

All groups almost exhibited high modularity *Q* across the sparsity range (0.1–0.3), showing a powerful modular structure of brain network organization (Supplementary Figure 1). We further conducted the following analyses on functional networks constructed at the 20% sparsity threshold. We identified four modules that corresponded to DMN, ECN, sensorimotor network (SMN) and visual network (VN) detected from group-averaged brain networks (Figure 1).

### Module-Wise Alterations and its Relationship With Cognition

**Figure 2:**
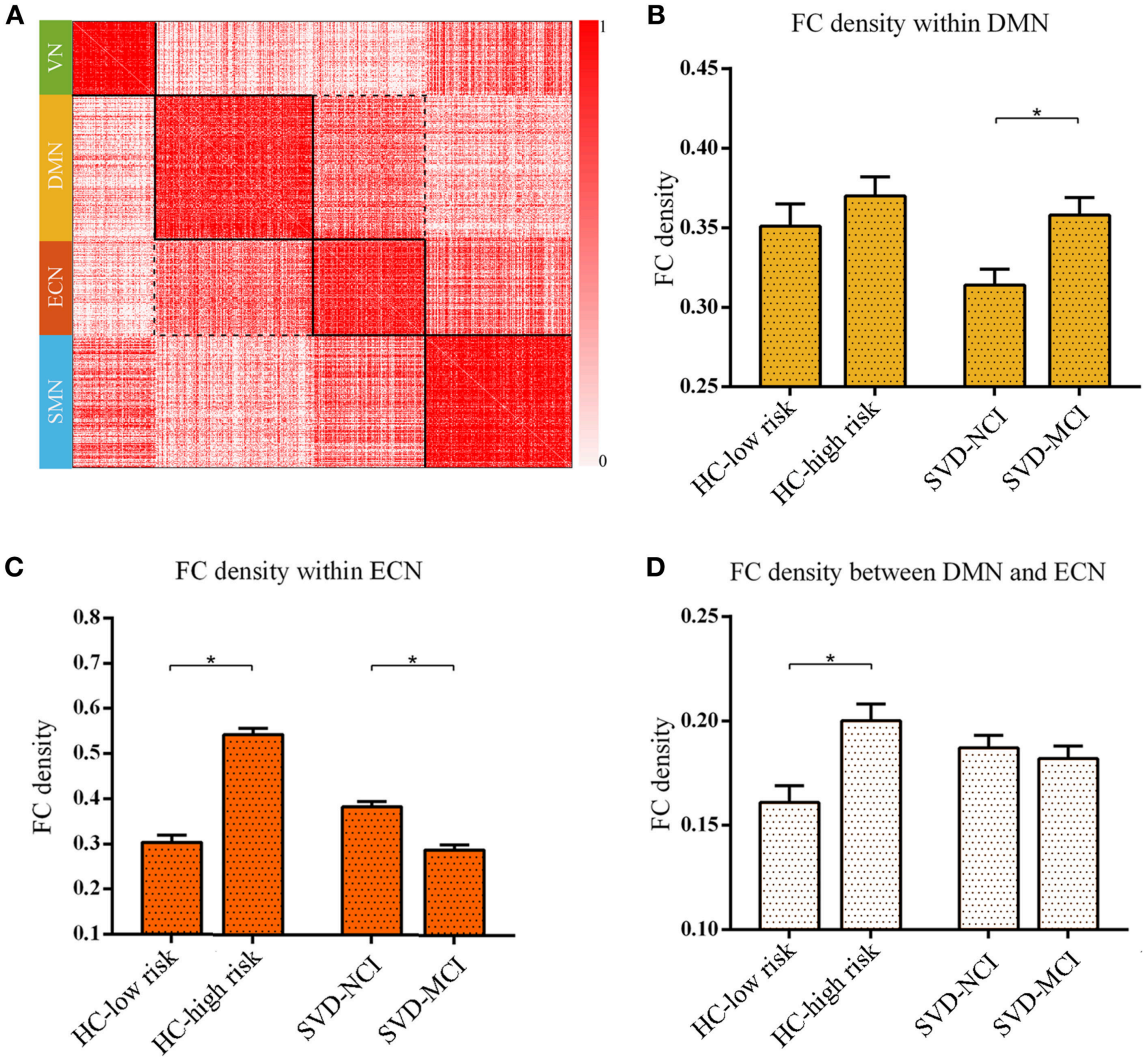
The reorganized pattern of intramodule and intermodule connectivity density within/between DMN and ECN. **(A)** The matrix showed the four modules and interactions between these modules. The darker color mean the higher connectivity density (uncorrected). **(B)** The FC density within DMN in SVD-MCI was significantly higher than it in SVD-NCI (^*^p = 0.004). **(C)** The FC density within ECN increased in HC-high risk compared with HC-low risk (^*^p < 0.001), whereas it significantly decreased in SVD-MCI compared with SVD-NCI (^*^p < 0.001). **(D)** The FC density between DMN and ECN in HC-high risk showed an higher pattern than it in HC-low risk (^*^p = 0.002). HC, healthy control; SVD, small vessel disease; NCI, non-cognitive impairment; MCI, mild cognitive impairment; DMN, default mode network; ECN, executive control network; SMN, sensorimotor network; VN, visual network; FC, functional connectivity.

We found the significant differences of intra-module connectivity density within DMN among the four groups (*F* = 4.919*, p* = 0.004) (Figure 2A). The further analysis indicated that SVD-MCI exhibited higher connectivity density than SVD-NCI (*p* = 0.004), while there was no significant difference between HC-low risk and HC-high risk (Figure 2B). Moreover, we found that IPS was positively associated with functional connectivity density within DMN (β = 0.501, *P* = 0.022) in SVD-NCI (Supplementary Figure 2A).

The group differences of intra-module connectivity density within ECN was also observed (*F* = 66.169*, p* < 0.001). In the HC group, the functional connectivity density within ECN remarkably enhanced with the increase of risk for SVD (*p* < 0.001), whereas in SVD group, the functional connectivity density notably decreased as the appearance of cognitive decline (*p* < 0.001) (Figure 2C). Multiple regression analyses indicated that IPS was negatively related to functional connectivity density within ECN (β = –0.432*, P* = 0.036) in SVD-NCI (Supplementary Figure 2B).

The inter-module connectivity density between DMN and ECN significantly differed in four groups (*F* = 3.671*, p* = 0.016). The HC-high risk group showed the more closely connected coupling between DMN and ECN compared with the HC-low risk group (*p* = 0.002) (Figure 2D). In contrast, there was no statistical difference between SVD groups. The FC density between DMN and ECN correlated negatively with DST (β = –0.587*, P* = 0.006) in HC-high risk (Supplementary Figure 2C). The alteration pattern of SMN and VN could be seen in Supplementary Figure 6.

### Nodal-Wise Alterations and its Relationship With Cognition

**Figure 3:**
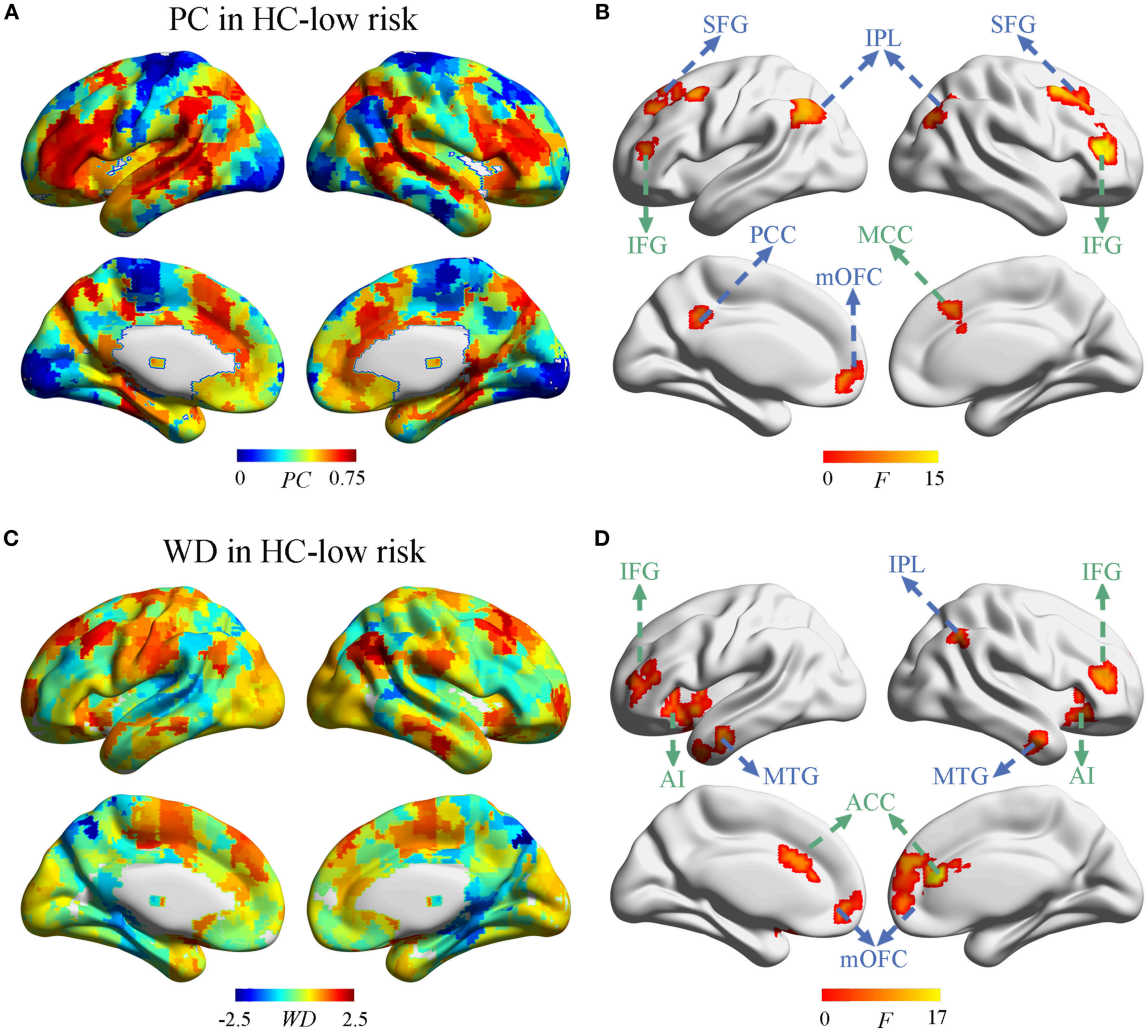
The distribution of PC and WD in the whole brain. **(A)** The PC distribution in HC-low risk. **(B)** Significant effects of vascular burden on PC were observed in the DMN (such as bilateral superior frontal gyrus [SFG], inferior parietal lobule [IPL], and left posterior cingulate cortex [PCC], medial orbitofrontal cortex [mOFC]) and the ECN (such as bilateral inferior frontal gyrus [IFG] and right midcingulate cortex [MCC]) (*P* < 0.01, FDR corrected). **(C)** The WD distribution in HC-low risk. **(D)** The WD was significantly regulated in the regions of DMN (such as bilateral mOFC, middle temporal gyrus [MTG], and the right IPL) and the ECN (such as bilateral ACC, IFG, and anterior insula [AI]) (*P* < 0.01, FDR corrected). HC, healthy control; PC, participant coefficient; WD, within module degree.

Next, we investigate whether and how the node properties within DMN and ECN were altered in SVD. The spatial distribution of PC and WD in group-averaged network were shown in Figure 3 and Supplementary Figure 3. Significant effects of vascular burden on PC were observed in the DMN (such as bilateral superior frontal gyrus [SFG], inferior parietal lobule [IPL], and left posterior cingulate cortex [PCC], medial orbitofrontal cortex [mOFC]) and the ECN (such as bilateral inferior frontal gyrus [IFG] and right midcingulate cortex [MCC]) (*P* < 0.01, FDR corrected) (Figure 3B). The *post hoc* tests revealed that PC in the DMN mostly tended to increase in subjects at high risk and decrease in SVD-MCI, whereas the alterations of PC in the ECN showed the increased pattern in SVD-MCI (Supplementary Figure 4). Interestingly, PC in the left mOFC (i.e., anterior DMN [aDMN]) only exhibited the increased pattern in SVD-MCI (Supplementary Figure 4A).

Group comparisons revealed that the WD was significantly regulated in the regions of the DMN (such as bilateral mOFC, middle temporal gyrus [MTG], and the right IPL) and the ECN (such as bilateral ACC, IFG, and anterior insula [AI]) (*P* < 0.01, FDR corrected) (Figure 3D). The *post-hoc* tests determined that WD did not homogeneously change within DMN and ECN. WD in the right IPL (i.e., pDMN) tended to increase, while WD in the bilateral mOFC and MTG (i.e., aDMN) decreased in SVD-MCI (Supplementary Figure 5A). In the ECN, WD of the bilateral IFG showed the similar pattern with the ECN module, whereas WD in bilateral ACC and AI had the increased tendency in SVD-MCI (Supplementary Figure 5B). We further found that WD of the right IPL negatively correlated with IPS (β = –0.494*, P* = 0.030) in HC-high risk (Supplementary Figure 2D). In SVD-MCI, WD of left AI was positively associated with IPS (β = 0.410*, P* = 0.028) (Supplementary Figure 2E).

### Right IFG Mediates PWMH-Induced Visuospatial Function Decline

**Figure 4:**
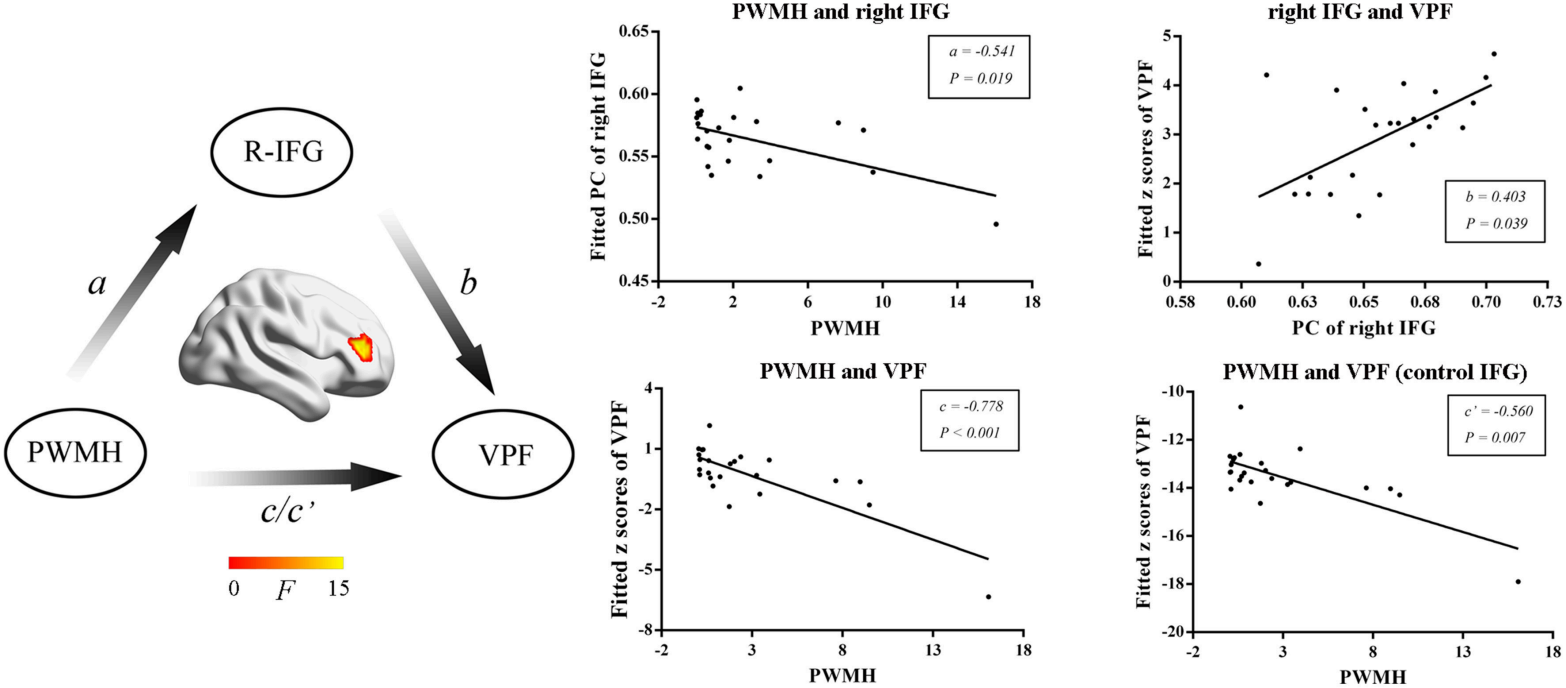
The mediation analyses in SVD-MCI. The PWMH was associated with PC in the right IFG (*a* = −0.541*, P* = 0.019) and VPF (*c* = −0.778*, P* < 0.001*; c*′ = −0.560*, P* = 0.007) and PC in the right IFG was related to VPF (*b* = 0.403*, P* = 0.039). PWMH, periventricular-white matter hyperintensities; IFG, inferior frontal gyrus; VPF, visuospatial processing function; PC, participant coefficient.

We then further investigated the relationship among MRI markers, network metrics and cognitive performance. WMH volumes, PWMH volumes, DWMH volumes, and numbers of lacunes were selected as MRI markers for further mediation analysis. In SVD-NCI, the PWMH positively correlated with PC in the left PCC (β = 0.449*, P* = 0.001). In SVD-MCI, the mediation analysis suggested that the PWMH was associated with PC in the right IFG (*a* = –0.541*, P* = 0.019) and VPF (*c* = –0.778*, P* < 0.001*; c*′ = –0.560*, P* = 0.007) and PC of right IFG was related to VPF (*b* = 0.403, *P* = 0.039) (Figure 4).

## Discussion

This study used graph-theoretical modularity for the first time to indicate that: (1) there was a high FC density in both the inter- and intra-network of the ECN and the DMN in the high risk individuals for SVD; (2) SVD-MCI patients showed a disrupted ECN intra-network and increased DMN connections; and (3) inter-module connectivity of the left IFG mediated the relationship between PWMH and visuospatial processing. These findings have important implications for the further understanding of the neural mechanism of SVD-related cognitive deficits.

Firstly, we wondered whether and how the brain modular architecture was altered in a population at high risk for SVD. Both DMN and ECN networks were chosen. The DMN (deactivated during tasks) is primarily involved in episodic memory and self-monitoring processing, while the ECN (activated during tasks) engages in the mediation of working memory, cognitive control and decision making. At the module level, our results revealed that connectivity density within the ECN increased in a high-risk population for SVD, but it did not within the DMN, which indicated that ECN (frontoparietal network) may be more susceptible to the vascular burden than the DMN and elucidated that the ECN supported cognitive processes by increasing its own integration (11). We also found increased intermodule connectivity density between the DMN and ECN that correlated negatively with DST (subcomponent of executive function), suggesting that the modular organization could increase flexibility and facilitate adaptation in response to environmental changes (8). By the evolutionary computation approach, hyperconnectivity between the DMN and ECN during recovery from traumatic brain injury reflected positive functional plasticity (26).

Next, we investigated the brain functional network of SVD-MCI patients. The results showed that the functional connectivity density within the ECN was significantly decreased. This hints that the frontoparietal network was particularly vulnerable to SVD-related damages, and SVD could hamper network function and impair cognition via a “disconnection syndrome” (27, 28). A combined functional and structural imaging study indicated that disrupted functional connectivity in the frontoparietal network mediated the impact of reduced white matter integrity in the bilateral superior longitudinal fasciculus on executive dysfunction in hypertensive patients with WMH (29). These functional alterations were closely associated with WMH and specific neuropsychological deficits.

Furthermore, increased functional connectivity within the DMN happened in SVD-MCI, which was positively associated with IPS. These findings may also reflect that the DMN and ECN played distinct roles in the progression of SVD, in which the ECN had a compensatory effect in the early stage of disease, and the DMN played a compensatory role in the late stage. The differential associations of DMN and ECN on cognition performance were also observed in other diseases. A resting-state fMRI study demonstrated that depressed participants showed decreased connectivity in the ECN and increased connectivity in the DMN compared to non-depressed participants and that these distinctive patterns of connectivity were associated with worse cognitive performance. In more detail, functional connectivity within the ECN was negatively associated with episodic memory performance while connectivity within the DMN was positively associated with episodic memory performance in the non-depressed participants (30). This highlights the potential importance of the DMN and ECN to adapt upon cognitive demands at different stages of the disease.

Excitingly, we found that some nodes, such as the bilateral ACC, AI, IFG, and right MCC within the ECN, exhibited increased intra- and inter-module functional connectivity in patients with SVD-MCI. However, regression analysis revealed that only the intra-module connectivity of the left AI was positively associated with IPS. Notably, we observed that, at the nodal level, PC and WD did not homogeneously change across the DMN; regions of the aDMN showed increased inter-module connectivity, whereas regions of the pDMN exhibited increased intra-module connectivity in SVD-MCI. Acutely, the aDMN is often involved in perception or self-referential processing, and the pDMN is more commonly related to episodic memory retrieval (31). Based on modularity analysis, the pDMN exhibited decreased intra-module connectivity in the apolipoprotein E ε4 carriers compared to that in the noncarriers, but the aDMN showed no significant alterations (10). Patients with schizophrenia showed increased posterior and decreased anterior connectivity within the DMN compared with healthy controls (32). Notwithstanding, the neurobiological mechanism behind the differentiated pattern requires further investigation. Overall, our results suggested that patients with SVD-MCI displayed complicated modular interactions with a parallel pattern of disruption and compensation in the ECN and DMN.

To further explore the relationship between vascular burden, network metrics and cognitive performance, mediation analysis was applied. The result suggested that PWMH induced VPF dysfunction regulated by the right IFG. VPF has been proposed to be susceptible to age-related decline and is preferentially disrupted in normal aging (33, 34). The visual processing-related regions can be divided into ventral and dorsal streams. The dorsal stream is involved in three major pathways, including the parieto-prefrontal, parieto-medial temporal, and parieto-premotor pathways (35). The parieto-prefrontal pathway is an important component of the dorsal stream in visuospatial processing (35). It sends input to the dorsal prefrontal region, which is essential for top-down executive control in visuospatial processing (35). The right IFG may play a central role in promoting the global processing of visuospatial perception (36). During the visuospatial working memory task, the fractional anisotropy and axial diffusivity of the white matter bundles connecting the IFG and fusiform were associated with processing speed (37). In subjects with autism spectrum disorder, poorer VPF was correlated with a disrupted white matter microstructure in the right inferior fronto-occipital fasciculus (38). Additionally, most of the investigations have revealed that the increasing burden of PWMH, not DWMH, may play an independent role in the decline of cognition (39). This evidence further supports our result that PWMH could result in the decline of visuospatial processing mediated by prefrontal functional connectivity in SVD.

Several issues in our study need to be noted. First, as a cross-sectional study, the data could not directly elucidate the relationship between imaging characteristics and SVD-related performance. Therefore, it is necessary to replicate our findings in future longitudinal studies. Second, the connectivity within/between different modules was binary undirected matrices. Thus, weighted matrices might provide more detailed information about network alterations. Third, our functional data preprocessing steps included global signal regression and we were only concerned with the positive correlations in the subsequent analyses. Further exploration of the effect of non-global signal regression and negative correlations on modular alterations in SVD participants is needed. Fourth, several modularity algorithms are currently available with different advantages. Different algorithms need to estimate the repeatability of our results. Finally, we only examined functional brain networks in the current study. It might be worth applying multimodal imaging techniques (e.g., arterial spin labeling) to explore the correlation between structural and functional networks.

## Conclusion

The modular architecture showed an altered reconfiguration pattern within/between the DMN and ECN and might have a mediation effect during the progression of SVD. These observations may provide novel insight into the underlying neural network mechanism of cerebral SVD-related cognitive impairment.

## Author Contributions

YX: conceived and designed the experiments. RQ, YG, XC, XW, JZ, and YJ: performed the experiments. RL, HC, and WL: analyzed the data. FB and BZ: contributed materials/analysis tools.

### Conflict of Interest Statement

The authors declare that the research was conducted in the absence of any commercial or financial relationships that could be construed as a potential conflict of interest.

## Supplementary Material

Supplementary Figure 1 The modularity *Q* across the sparsity range (0.1–0.3) in each group. HC, healthy control; SVD, small vessel disease; NCI, non-cognitive impairment; MCI, mild cognitive impairment.

Supplementary Figure 2 The significant relationship between modular indexes and cognitive assessments. **(A)** IPS was positively associated with functional connectivity density within DMN (β = 0.501, *P* = 0.022) in SVD-NCI. **(B)** IPS was negatively related to functional connectivity density within ECN (β = −0.432, *P* = 0.036) in SVD-NCI. **(C)** The FC density between DMN and ECN correlated negatively with DST (β = −0.587, *P* = 0.006) in HC-high risk. **(D)** WD of the right IPL negatively correlated with IPS (β = −0.494, *P* = 0.030) in HC-high risk. **(E)** WD of left AI was positively associated with IPS (β = 0.410, *P* = 0.028) in SVD-MCI. HC, healthy control; SVD, small vessel disease; NCI, non-cognitive impairment; MCI, mild cognitive impairment; WD, within module degree; FC, functional connectivity; DMN, default mode network; ECN, executive control network; IPS, information processing speed; DST, digit span test; IPL, inferior parietal lobe; AI, anterior insula.

Supplementary Figure 3 The distribution of PC and WD in HC-high risk, SVD-NCI, and SVD-MCI. HC, healthy control; SVD, small vessel disease; NCI, non-cognitive impairment; MCI, mild cognitive impairment; PC, participant coefficient; WD, within module degree.

Supplementary Figure 4 The *post-hoc* tests of PC in significantly differentiated brain regions involved in DMN **(A)** and ECN **(B)**. HC, healthy control; SVD, small vessel disease; NCI, non-cognitive impairment; MCI, mild cognitive impairment; PC, participant coefficient; SFG, superior frontal gyrus; IPL, inferior parietal lobe; PCC, posterior cingulate cortex; mOFC, medial orbitofrontal cortex; IFG, inferior frontal gyrus; MCC, right midcingulate cortex.

Supplementary Figure 5 The *post-hoc* tests of WD in significantly differentiated brain regions involved in DMN **(A)** and ECN **(B)**. HC, healthy control; SVD, small vessel disease; NCI, non-cognitive impairment; MCI, mild cognitive impairment; WD, within module degree; MTG, middle temporal gyrus; IPL, inferior parietal lobe; mOFC, medial orbitofrontal cortex; IFG, inferior frontal gyrus; ACC, anterior cingulate cortex; AI, anterior insula.

Supplementary Figure 6 FC density within the visual network and sensorimotor network. **(A)** The FC density within the visual network showed a significant group difference (*p* = 0.041, ANOVA, controlled age, sex, and years of education). The FC density within the visual network in HC-high risk was significantly decreased compared to HC-low risk (*p* = 0.005). **(B)** There was no significant difference in FC density within the sensorimotor network. FC, functional connectivity; HC, healthy control; CSVD, cerebral small vessel disease; NCI, non-cognitive impairment; CI, cognitive impairment.
