# Supplementary figures and images for: The Altered Reconfiguration Pattern of Brain Modular Architecture Regulates Cognitive Function in Cerebral Small Vessel Disease
Source: Front Neurol. 2019 Apr 5;10:324. doi: 10.3389/fneur.2019.00324 (PMC6461194; doi:10.3389/fneur.2019.00324)

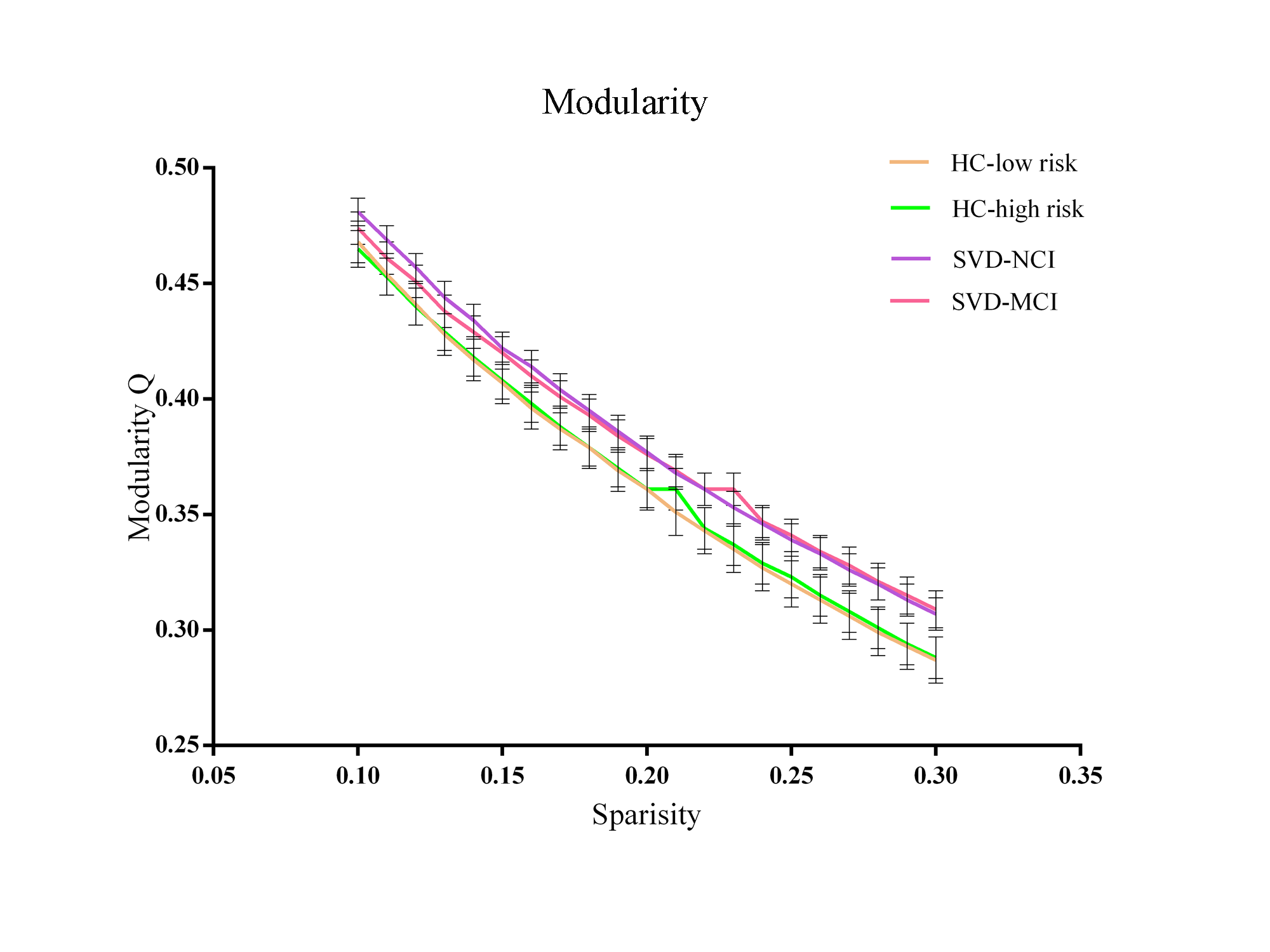

Supplement: Supplementary Figure 1 — The modularity Q across the sparsity range (0.1–0.3) in each group. HC, healthy control; SVD, small vessel disease; NCI, non-cognitive impairment; MCI, mild cognitive impairment. [file Image_1.TIF]

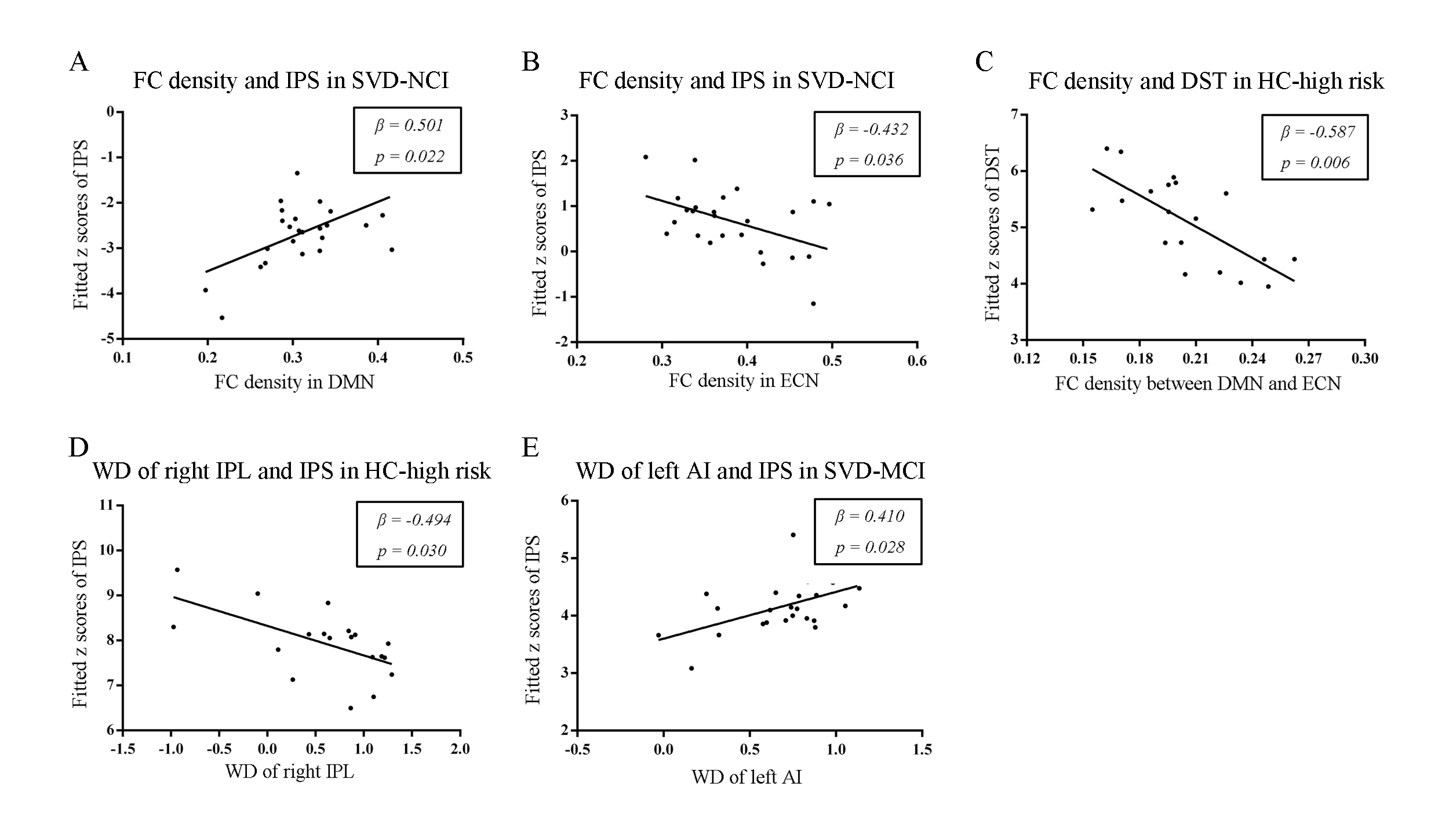

Supplement: Supplementary Figure 2 — The significant relationship between modular indexes and cognitive assessments. (A) IPS was positively associated with functional connectivity density within DMN (β = 0.501, P = 0.022) in SVD-NCI. (B) IPS was negatively related to functional connectivity density within ECN (β = −0.432, P = 0.036) in SVD-NCI. (C) The FC density between DMN and ECN correlated negatively with DST (β = −0.587, P = 0.006) in HC-high risk. (D) WD of the right IPL negatively correlated with IPS (β = −0.494, P = 0.030) in HC-high risk. (E) WD of left AI was positively associated with IPS (β = 0.410, P = 0.028) in SVD-MCI. HC, healthy control; SVD, small vessel disease; NCI, non-cognitive impairment; MCI, mild cognitive impairment; WD, within module degree; FC, functional connectivity; DMN, default mode network; ECN, executive control network; IPS, information processing speed; DST, digit span test; IPL, inferior parietal lobe; AI, anterior insula. [file Image_2.TIF]

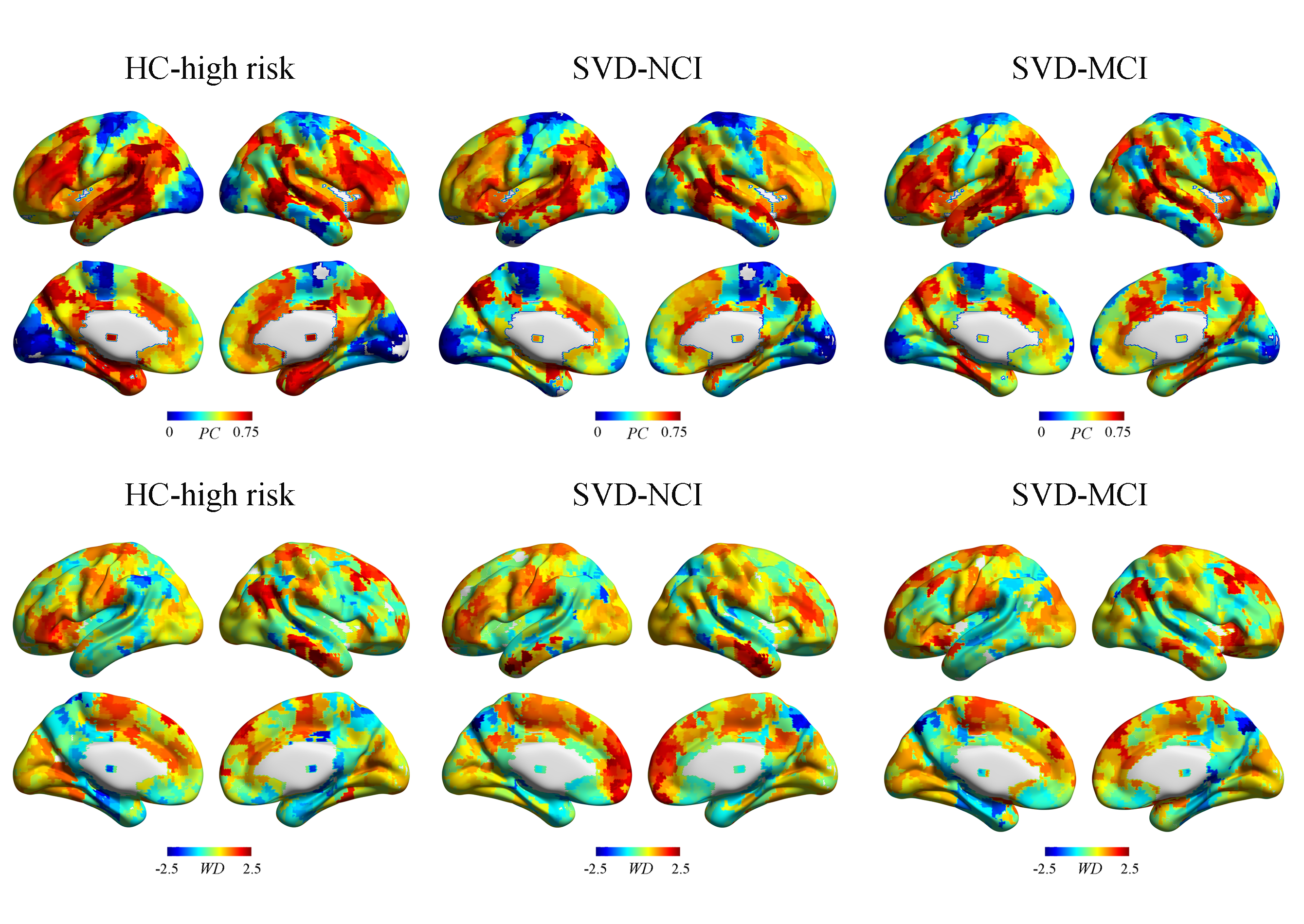

Supplement: Supplementary Figure 3 — The distribution of PC and WD in HC-high risk, SVD-NCI, and SVD-MCI. HC, healthy control; SVD, small vessel disease; NCI, non-cognitive impairment; MCI, mild cognitive impairment; PC, participant coefficient; WD, within module degree. [file Image_3.TIF]

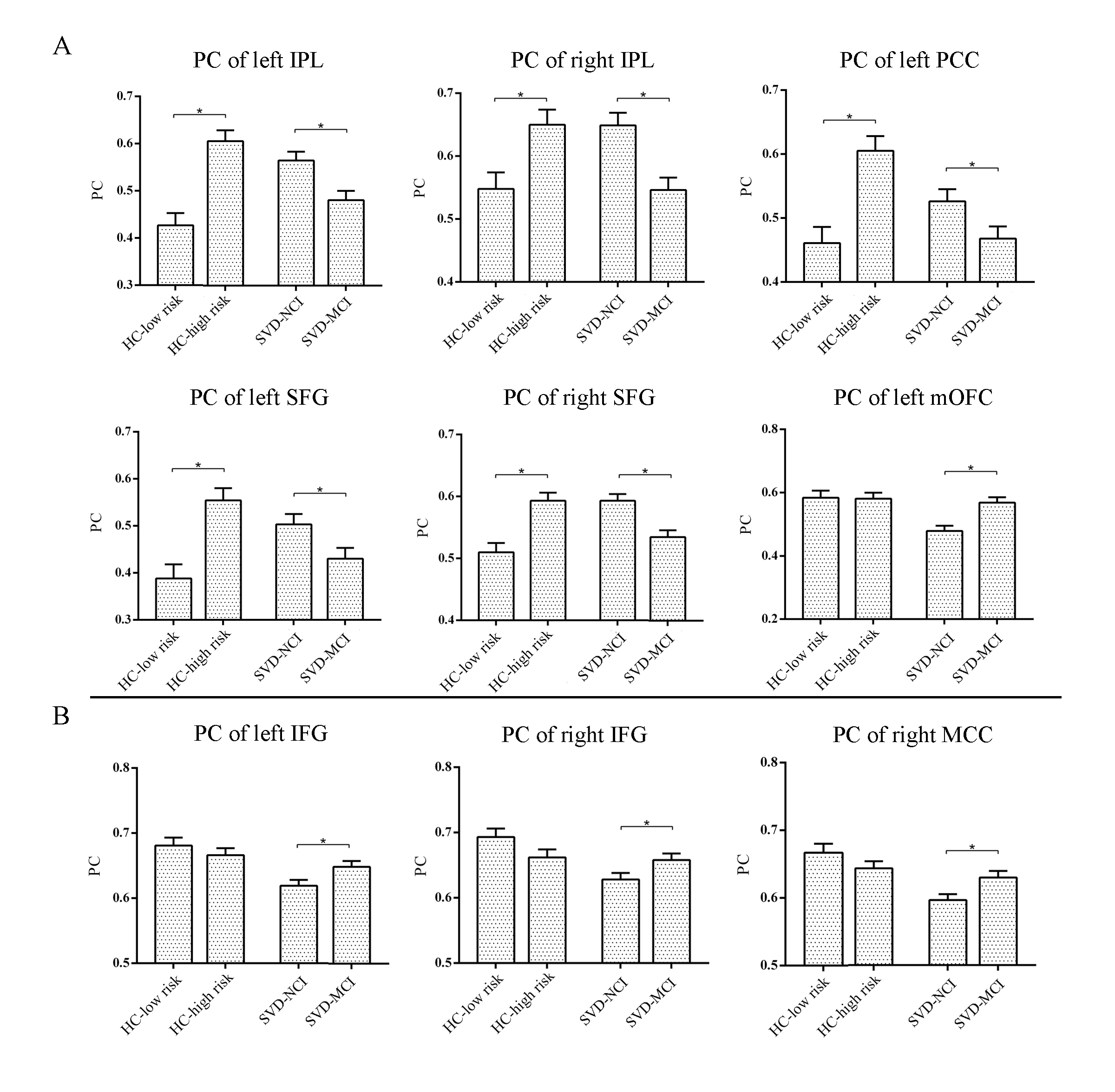

Supplement: Supplementary Figure 4 — The post-hoc tests of PC in significantly differentiated brain regions involved in DMN (A) and ECN (B). HC, healthy control; SVD, small vessel disease; NCI, non-cognitive impairment; MCI, mild cognitive impairment; PC, participant coefficient; SFG, superior frontal gyrus; IPL, inferior parietal lobe; PCC, posterior cingulate cortex; mOFC, medial orbitofrontal cortex; IFG, inferior frontal gyrus; MCC, right midcingulate cortex. [file Image_4.TIF]

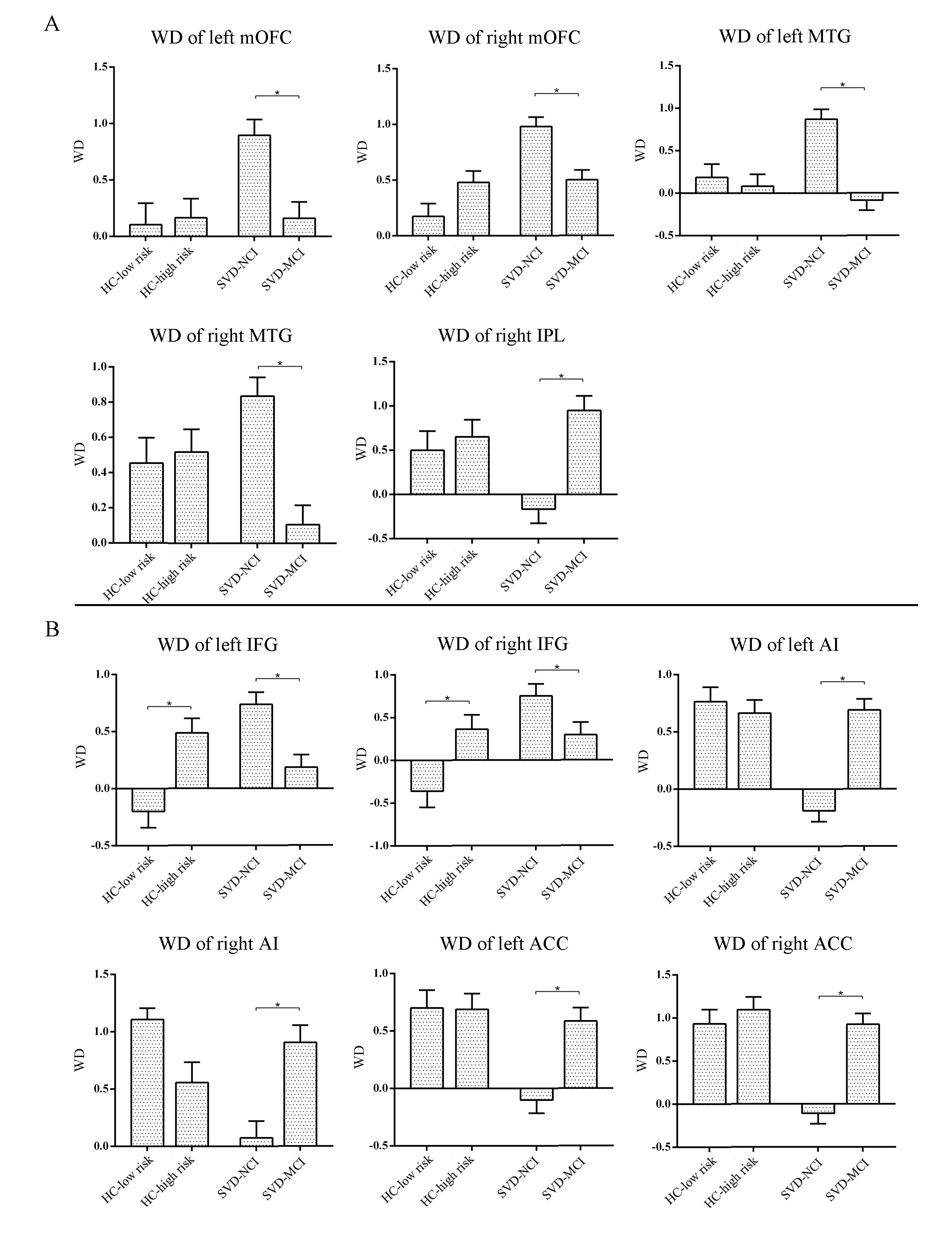

Supplement: Supplementary Figure 5 — The post-hoc tests of WD in significantly differentiated brain regions involved in DMN (A) and ECN (B). HC, healthy control; SVD, small vessel disease; NCI, non-cognitive impairment; MCI, mild cognitive impairment; WD, within module degree; MTG, middle temporal gyrus; IPL, inferior parietal lobe; mOFC, medial orbitofrontal cortex; IFG, inferior frontal gyrus; ACC, anterior cingulate cortex; AI, anterior insula. [file Image_5.TIF]

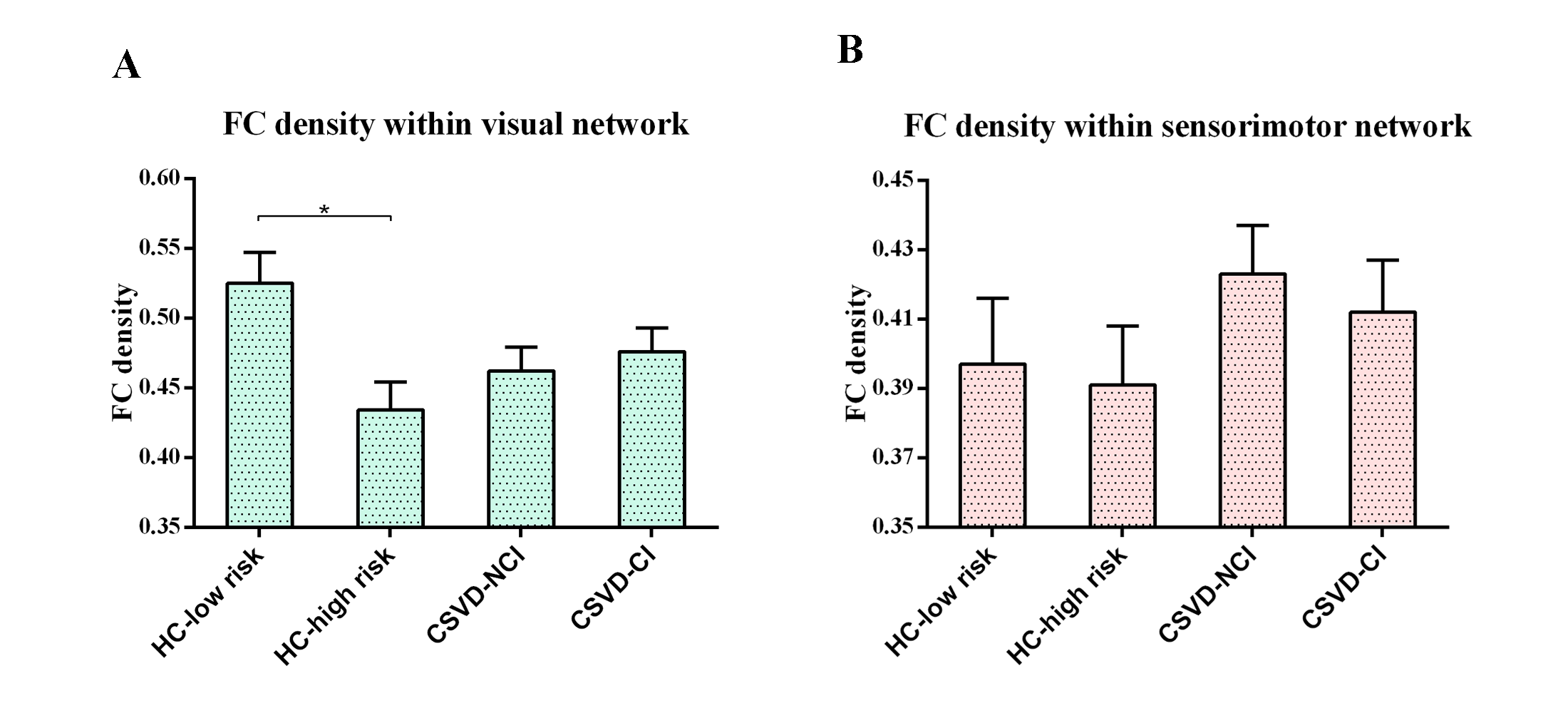

Supplement: Supplementary Figure 6 — FC density within the visual network and sensorimotor network. (A) The FC density within the visual network showed a significant group difference (p = 0.041, ANOVA, controlled age, sex, and years of education). The FC density within the visual network in HC-high risk was significantly decreased compared to HC-low risk (p = 0.005). (B) There was no significant difference in FC density within the sensorimotor network. FC, functional connectivity; HC, healthy control; CSVD, cerebral small vessel disease; NCI, non-cognitive impairment; CI, cognitive impairment. [file Image_6.TIF]
